# Original rDNA Semaglutide Versus Synthetic Copies: Differences and Clinical Implications

**DOI:** 10.7759/cureus.114959

**Published:** 2026-08-21

**Authors:** Viswanathan Mohan, Sanjay Kalra, Abhay Ahluwalia, Kalyan K Gangopadhyay, Arpandev Bhattacharyya, Banshi Saboo, Jothydev Kesavadev, Santosh Ramakrishnan, Om J Lakhani, Lakshmi Nagendra, Abhay Kumar Sahoo, Ameya Joshi, Nagesh V. Sri, Shashank R Joshi

**Affiliations:** 1 Diabetes and Endocrinology, Madras Diabetes Research Foundation (ICMR Centre of Excellence in Research) and Dr. Mohan’s Diabetes Specialities Centre (IDF Centre of Excellence in Diabetes Care), Chennai, IND; 2 Endocrinology, Bharti Hospital, Karnal, IND; 3 Endocrinology, Fortis Memorial Research Institute, Gurgaon, IND; 4 Endocrinology, Peerless Hospitals, Kolkata, IND; 5 Endocrinology, Manipal Hospitals, Old Airport Road, Bangalore, IND; 6 Diabetology, Diacare - Diabetes Care and Hormone Clinic, Ahmedabad, IND; 7 Diabetology, Jothydev’s Diabetes Research Centre, Thiruvananthapuram, IND; 8 Endocrinology, Magna Centres for Obesity Diabetes and Endocrinology, Hyderabad, IND; 9 Endocrinology, AB Plus Hospital, Ahmedabad, IND; 10 Endocrinology, JSS Medical College, JSS Academy of Higher Education and Research, Mysuru, IND; 11 Endocrinology, Apollo Hospital, Bhubaneshwar, IND; 12 Endocrinology, Diabetes and Endocrine Clinics, Bhaktivedanta Hospital and Research Institute, Karuna Hospital, Mumbai, IND; 13 Endocrinology, Sri Nagesh Diabetes, Thyroid and Endocrine Clinic, Hyderabad, IND; 14 Diabetology and Endocrinology, Lilavati Hospital and Research Centre, Mumbai, IND

**Keywords:** abcde framework, glp-1 receptor agonist, pharmacosimilar, semaglutide, synthetic peptide

## Abstract

The therapeutic dominance of semaglutide in managing diabetes, obesity, and cardio-kidney-metabolic diseases has spurred an influx of generics, biosimilars, and compounded *pharmacosimilars*. However, shared nomenclature does not guarantee therapeutic equivalence. This analysis introduces the ABCDE framework (author-proposed conceptual checklist) to differentiate reference r-DNA semaglutide from synthetic or informally marketed copies. Evaluation rests on five pillars: (A) approved status, where regulatory authorization does not inherently imply interchangeability; (B) bioavailability, noting that identical exposure metrics may mask divergent impurity profiles and immunogenic risks; (C) consistency, highlighting that recombinant versus synthetic manufacturing can alter peptide stability and aggregation; (D) drug-device performance, with standardized pens helping to mitigate dosing errors that may occur with multidose vials; and (E) evidence, emphasizing that the robust clinical data from the SUSTAIN/STEP/SELECT programs are not transferable to copies. Consequently, we suggest that clinicians may find it useful to weigh product-specific evidence over label similarity when evaluating pharmacosimilars, using the ABCDE domains as a conceptual checklist (author-proposed mnemonic). Until stronger product-specific comparability evidence is available for alternative products, many clinicians may reasonably view reference semaglutide as the best-characterized option.

## Editorial

Semaglutide has become an important therapeutic option across diabetes, obesity, and broader cardio-kidney-metabolic care (which involves interconnected disorders of the heart, kidneys, and metabolism). The regulator-approved original semaglutide products are identified in product information as recombinant DNA-derived medicines and are supported by a large, peer-reviewed evidence base. An annual report from the manufacturer stated that injectable semaglutide had been launched in more than 75 countries, and a 2026 company press release from the manufacturer stated that semaglutide had been used for more than 49 million patient-years across semaglutide products, supported by over 50 clinical trials and 10 real-world studies [1-3].

The exclusivity landscape is evolving, with earlier generic entry already underway in several markets, such as India, Canada, Brazil, and China. The widespread clinical use of semaglutide has attracted a widening field of alternatives, including branded copies, follow-on products, compounded preparations, and products marketed through less formal channels. The term *pharmaco-similar* may be used to describe both biosimilar and generic versions of original rDNA semaglutide. (*Pharmacosimilars* refers to an author-proposed descriptive term for generic, biosimilar, or informally marketed copies of reference semaglutide; it does not constitute a recognized regulatory category.) [4,5].

This expanding landscape introduces concerns beyond molecule-level comparability. These might include look-alike/sound-alike prescribing risks (a theoretical concern), differences in device presentation, variable counselling requirements (a theoretical concern), and uncertainty regarding in-use stability in multidose presentations. This makes it important to distinguish categories that are often conflated in routine discussion: (1) reference semaglutide products, supported by a large evidence base and regulated manufacturing controls, and (2) regulator-approved generics or pharmacosimilars, where available, that must demonstrate quality and, typically, bioequivalence or appropriate bridging in line with local regulatory pathways.

These concerns are especially relevant for injectable semaglutide because it is a drug-device product. In such products, real-world performance depends not only on the active molecule but also on dose-setting accuracy, storage conditions, repeated puncture integrity, and validated in-use stability. Accordingly, claims regarding extended in-use duration should be supported by product-specific evidence on chemical stability, physical stability, microbiological integrity, and device performance under simulated clinical conditions.

In this context, the key issue is not merely product naming but whether meaningful comparability has been demonstrated. Kalra et al. have proposed that such molecules be discussed under the broader concept of “pharmacosimilars,” an author-proposed term without regulatory standing, reflecting the need to evaluate them based on identity, manufacturing, device performance, and evidence rather than on sequence or label alone [4].

**Figure 1:**
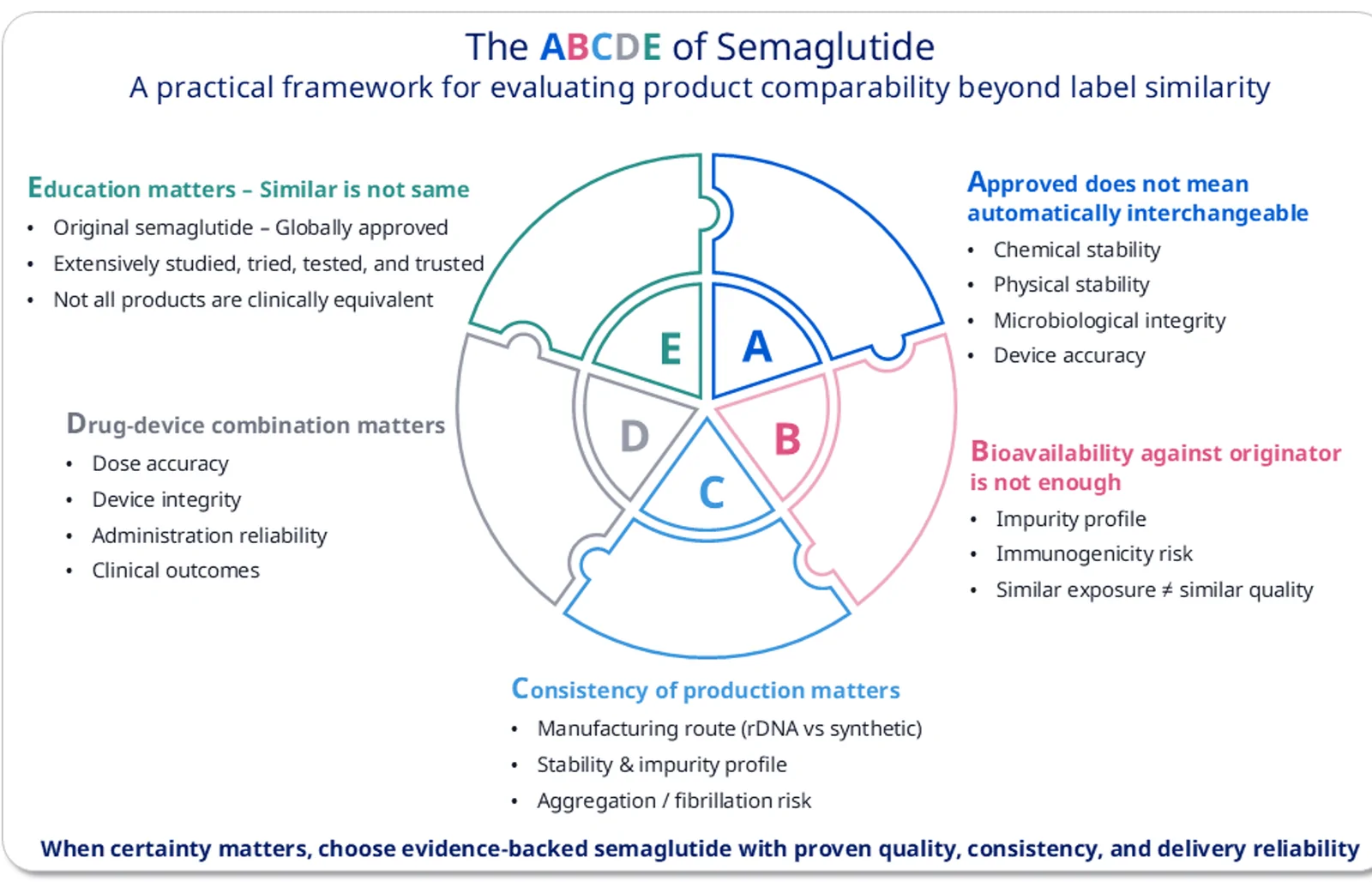
ABCDE framework for evaluating semaglutide product comparability beyond label similarity. ABCDE is an author-developed mnemonic and conceptual framework that has not been endorsed by any regulatory authority.

To simplify this discussion, we apply an ABCDE framework (ABCDE is an author-developed mnemonic and conceptual framework that has not been endorsed by any regulatory authority) for evaluating semaglutide products beyond label similarity alone:

This framework is intended to provide a practical and scientifically defensible structure for judging semaglutide products based on identity, quality, evidence, device reliability, and traceable distribution, rather than on naming similarity alone (Table 1, Figure 1) [1,6,7].

**Table 1:** ABCDE* framework for evaluating pharmacosimilars of reference semaglutide. ^*^ABCDE is an author-developed mnemonic and conceptual framework that has not been endorsed by any regulatory authority.

ABCDE framework	
A	Approved does not mean automatically interchangeable
B	Bioavailability against the originator may not be enough
C	Consistency of production matters
D	Drug-device combination matters
E	Education/Evidence matters because similar is not necessarily the same

A: Approved does not mean automatically interchangeable

For clarity, we use *regulatory approval* to mean market authorization under a given pathway; *therapeutic equivalence* to mean demonstrated similarity in clinical effect; and *interchangeability* to mean a regulatory determination that one product may be substituted for another without clinician oversight. These are distinct determinations, and the criteria for each differ across jurisdictions. For semaglutide, approval through an alternative regulatory pathway confirms compliance with that pathway’s requirements, but it does not by itself prove that the product can be substituted for the reference product without clinically meaningful differences. Regulatory approval should not automatically be interpreted as therapeutic interchangeability [8,9].

This principle is reflected in current regulatory thinking. The European Medicines Agency (EMA) guideline on synthetic peptides requires detailed attention to process definition, characterization, specifications, and analytical control, rather than assuming sameness on the basis of peptide sequence alone [1]. In India, the Central Drugs Standard Control Organization (CDSCO) has clarified that a synthetically manufactured drug previously approved as an rDNA-derived drug should be submitted as a subsequent new drug and evaluated under the New Drugs and Clinical Trials Rules, 2019, with consideration of safety and efficacy, rather than being treated as a routine interchangeable generic [10]. Together, these positions support a more precise conclusion: for semaglutide, approval may establish market authorization, but interchangeability requires stronger product-specific evidence on comparability and clinical use conditions [1]. The U.S. Food and Drug Administration (FDA) generally considers amino acid polymers composed of 40 or fewer amino acids to be peptides regulated under the Federal Food, Drug, and Cosmetic (FD&C) Act rather than proteins regulated under the Public Health Service Act. Semaglutide is a 31-amino-acid GLP-1 receptor agonist and therefore falls within this peptide classification. Consequently, appropriately characterized follow-on synthetic semaglutide products may be eligible for development through the Abbreviated New Drug Application (ANDA) pathway, subject to demonstration of active-ingredient sameness and other FDA requirements [11,12].

**Figure 2:**
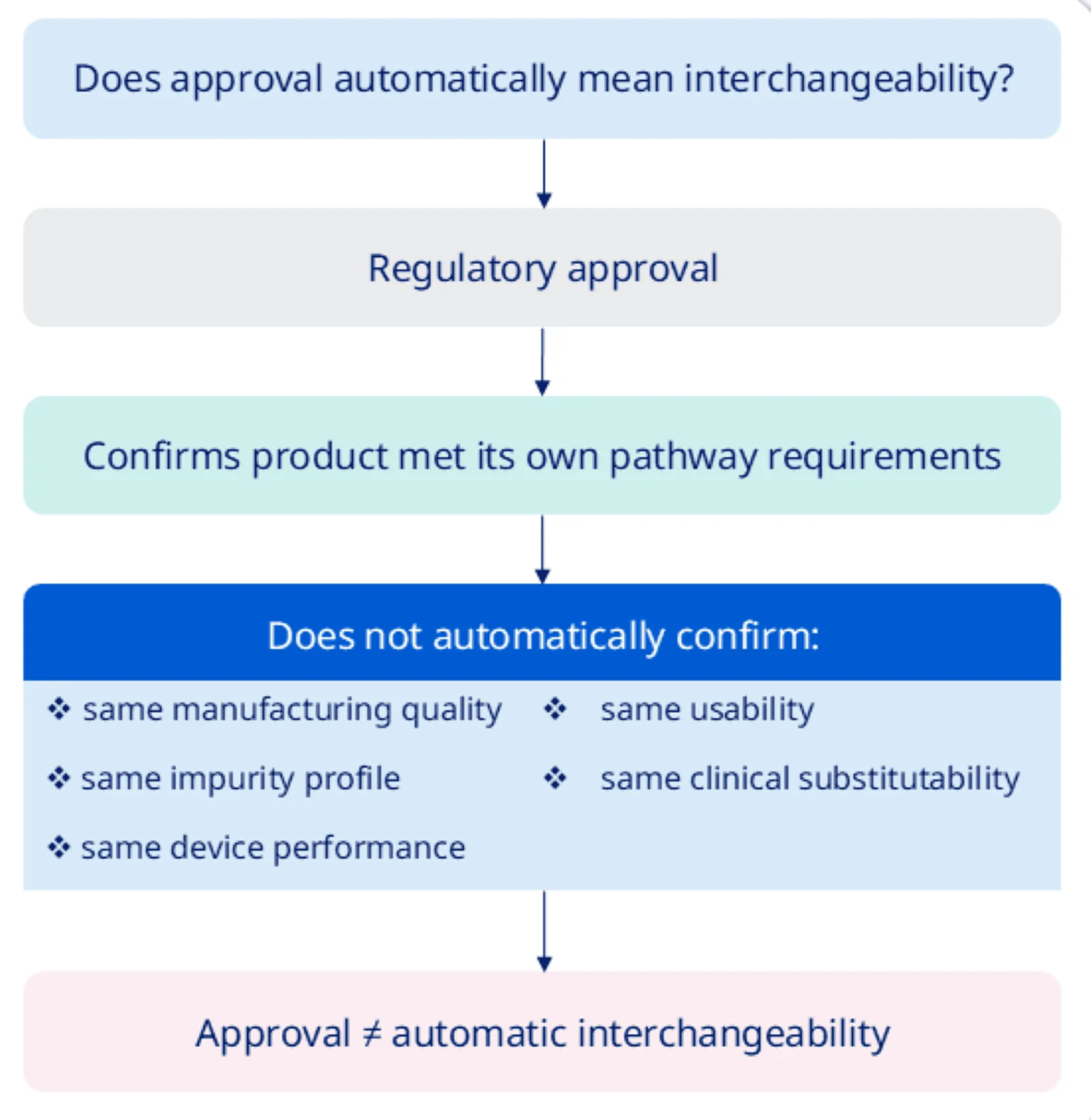
A - Approved does not mean automatically interchangeable. Created by the authors using Microsoft PowerPoint (Microsoft Corporation, Redmond, WA).

Non-originator semaglutide products should be clearly distinguished as regulator-approved generic/follow-on products, pharmacy-compounded preparations, and unauthorized or counterfeit products. These categories differ substantially in regulatory oversight, quality assurance, and potential safety risks; therefore, evidence relating to compounded or unauthorized products should not be extrapolated to regulator-approved generics. For injectable semaglutide, the issue is further complicated by the fact that the medicine is also a drug-device product. FDA-approved injectable semaglutide products are supplied in standardized prefilled pens, whereas non-standard presentations may shift dose measurement and administration burden to the user. The FDA has documented dosing errors with multidose vial presentations of compounded semaglutide, including some requiring hospitalization and involving five- to 20-fold overdoses, highlighting that device design and user interface are clinically relevant variables rather than peripheral details [6]. Accordingly, chemical stability, physical stability, microbiological integrity, and dose-delivery reliability are all relevant when judging whether one semaglutide product can genuinely be used in place of another (Figure 2) [6].

Clinical Implication

Regulatory approval may support market authorization, but interchangeability requires stronger product-specific evidence.

B: Bioavailability against originator may not be enough

Standard bioequivalence criteria (typically requiring the 90% confidence interval for the test/reference ratio of exposure metrics to fall within 80%-125%) are designed to bound pharmacokinetic differences, not to guarantee equivalent clinical outcomes. Whether a within-range difference in semaglutide exposure would materially change cardiovascular or other hard outcomes has not, to our knowledge, been directly tested for any follow-on semaglutide product; this is an evidence gap rather than a demonstrated risk, and we present it as such.

For synthetic peptides, regulatory and scientific evaluations extend beyond exposure metrics and must consider structural characterization, process-related and route-specific impurities, sequence variants and aggregates, stability behavior, and potential immunogenicity.

Why Does This Matter?

(1) These factors may influence safety, stability, and biological performance independent of exposure metrics.

(2) The EMA guideline on synthetic peptides specifically requires attention to manufacturing process, impurity profile, analytical control, and comparability considerations in addition to amino-acid sequence identity [1].

(3) In the study by Staby et al., differences in manufacturing process and scale affected impurity profile and physical stability in follow-on GLP-1 analog products [13].

(4) Several peptide-related impurities were predicted in silico to contain potential T-cell epitopes; although this represents a theoretical rather than clinically demonstrated immunogenicity signal, it cannot be ignored and warrants further experimental evaluation.

(5) Staby et al. therefore argued that follow-on liraglutide and semaglutide-related products should be clinically evaluated for efficacy and safety, rather than assumed to be comparable on analytical grounds alone [13].

**Figure 3:**
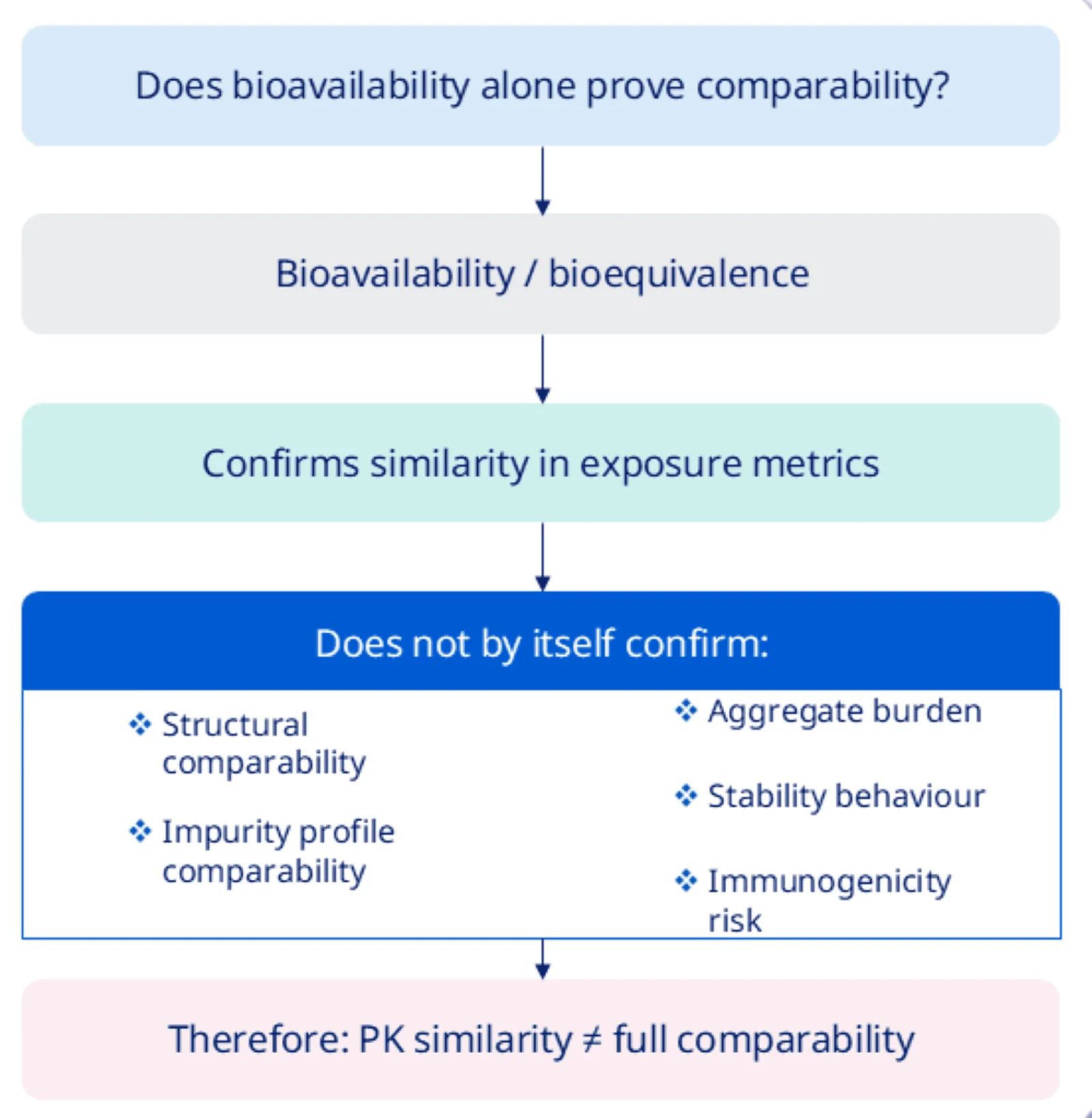
B - Bioavailability against the originator is not enough. Image created by the authors using Microsoft PowerPoint (Microsoft Corporation, Redmond, WA).

The immunogenicity point is especially important. Even for the originator semaglutide, anti-drug antibodies were specifically monitored throughout the phase 3 SUSTAIN programme: using a tiered antibody analysis, Staby et al. reported anti-semaglutide antibody positivity in 1.4% of semaglutide-treated patients, lower than that for liraglutide, with no neutralizing antibodies detected [13]. The low observed rate in the reference product is reassuring, but it should not be misused to imply that immunogenicity concerns can be dismissed for follow-on products with different impurity or aggregation profiles (Figure 3).

Clinical Implication

Bioavailability may be necessary, but it is not enough without supporting evidence on impurity profile, stability, and immunogenicity.

C: Consistency of production matters

For peptides like semaglutide, which occupy an intermediate space between conventional small-molecule generics and biologics, consistency of production is a key determinant of product quality because the process helps define the final product. The approved reference products describe semaglutide as a human GLP-1 analog produced in *Saccharomyces cerevisiae* cells using recombinant DNA technology [14-16].

In practice, this means that the active substance is generated through a controlled sequence of manufacturing steps, including upstream expression, recovery, purification, and subsequent modification steps such as acylation to attach the fatty diacid side chain.

Together, these steps help establish the final quality attributes of the product [14-16].

Why Does This Matter?

(1) Peptides occupy an intermediate space between small molecules and biologics.

(2) Different manufacturing routes can generate different impurity burdens and aggregation tendencies.

(3) Even with the same intended active sequence, differences in process may affect stability and consistency.

**Figure 4:**
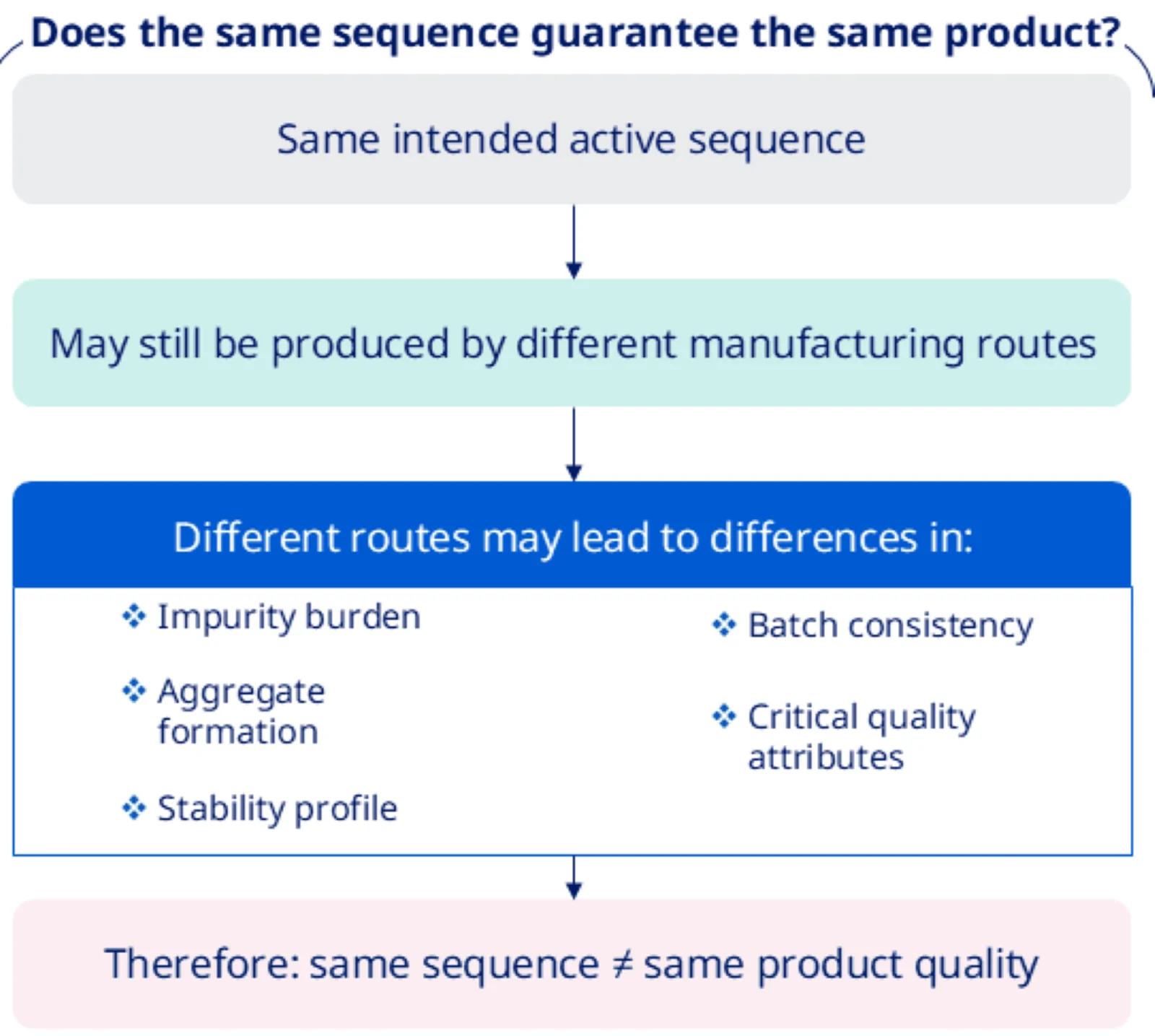
C - Consistency of production matters. Image created by the authors using Microsoft PowerPoint (Microsoft Corporation, Redmond, WA).

**Figure 5:**
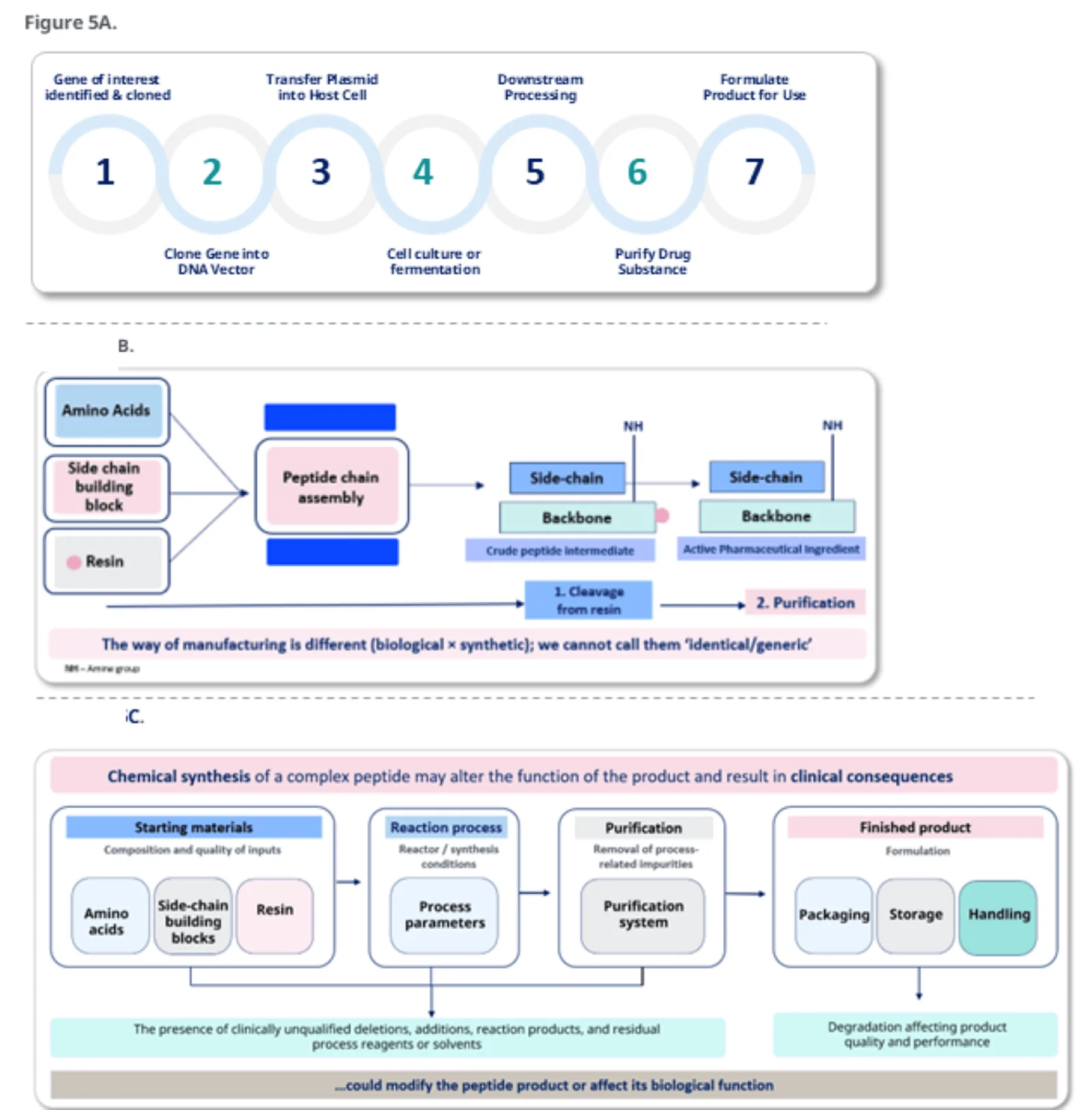
Comparison of recombinant and synthetic manufacturing routes and their implications for semaglutide product quality. Image created by the authors using Microsoft PowerPoint (Microsoft Corporation, Redmond, WA). (A) Recombinant DNA production pathway for semaglutide (the process defines the product). (B) Synthetic peptide production pathway for semaglutide. (C) Potential impact of process modification on semaglutide product quality and function.

Wu et al. emphasized that peptide drug products require control not only of assay and purity but also of manufacturing-related impurities, aggregates, and other quality attributes that may affect safety and efficacy [9]. Likewise, Vulto and Jaquez noted more broadly that, in complex biological products, critical quality attributes can vary according to the manufacturing process and therefore must be systematically characterized and controlled (Figures 4-5) [8].

Clinical Implication

Comparability cannot be inferred from label identity alone. Whether following a manufacturing change within the originator process or between products from different manufacturers, comparability must be supported by appropriate product-specific assessment of process consistency, impurity profile, stability, and other relevant quality attributes [13,17-19].

D: Drug-device combination matters

**Figure 6:**
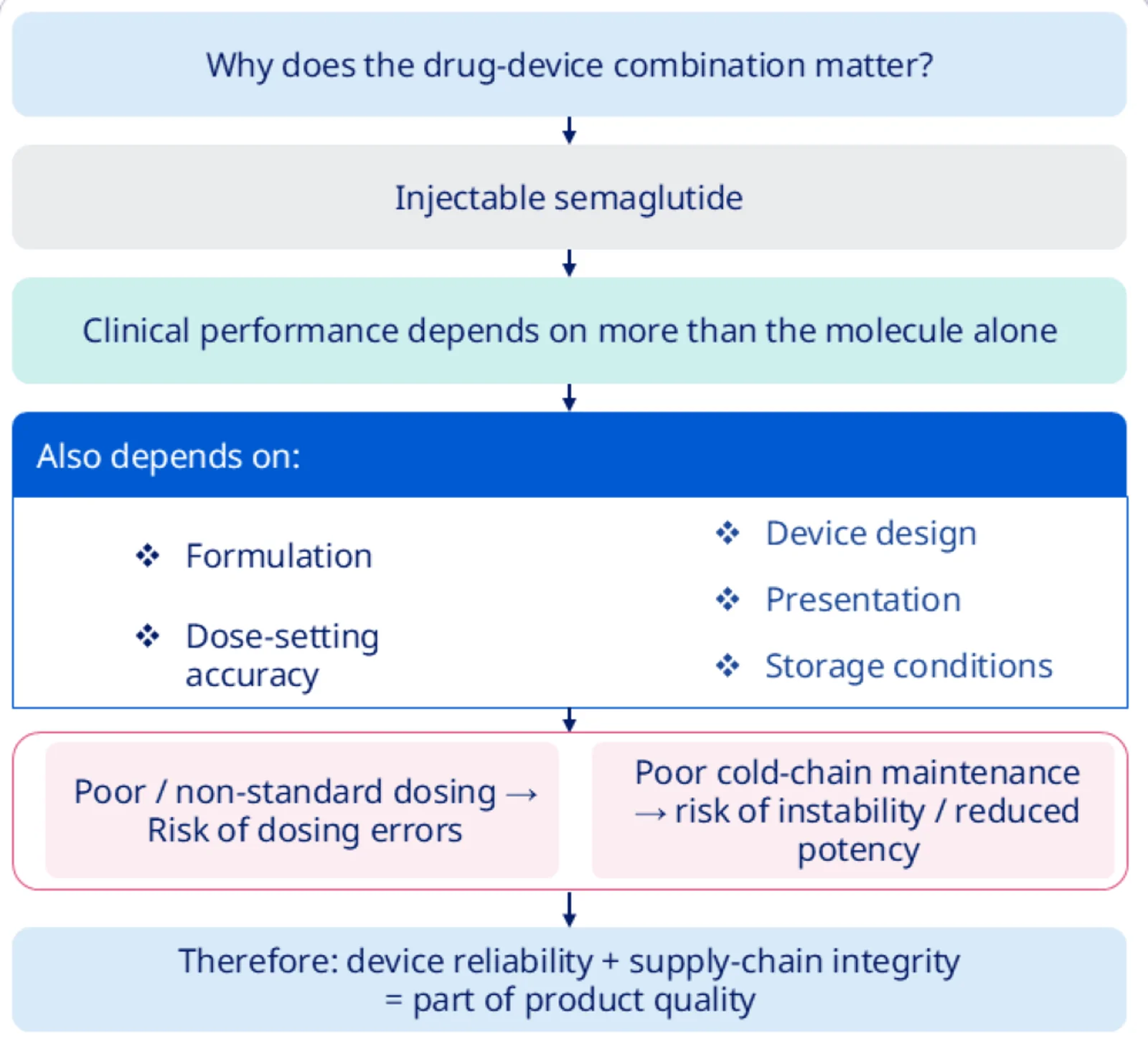
D - Drug-device combination matters. Image created by the authors using Microsoft PowerPoint (Microsoft Corporation, Redmond, WA).

For injectable semaglutide, the delivery system is an integral part of the medicinal product. Clinical performance depends not only on the molecule but also on formulation, device design, presentation, dose-setting accuracy, and storage conditions (Figure 6).

Why Does This Matter?

(1) The regulator-approved reference injectable semaglutide products are supplied in pre-filled pens with standardized concentrations and milligram-based dosing.

(2) Synthetic copies or non-standard preparations may be supplied in multi-dose vials with variable concentrations and unit-based instructions.

(3) This increases the risk of preventable dosing errors, especially when patients or caregivers must convert between milligrams, milliliters, and syringe units.

(4) Follow-on and compounded semaglutide is also marketed in multiple concentrations and, in some cases, in flexible-dose pens or vial-and-syringe formats; such presentations can further increase the risk of misdosing and associated adverse effects, particularly during dose-escalation or when patients switch between products [5].

(5) Higher-concentration formulations reduce injection volume, but formulation choices, including tonicity agents and preservatives, can influence local tolerability; in comparative studies of the originator product, changes in excipient composition were associated with differences in injection-site discomfort, underscoring that concentration and excipient selection are not clinically neutral [20].

(6) FDA has documented dosing errors specifically with multi-dose vial presentations of compounded semaglutide, including some requiring hospitalization and involving five- to 20-fold overdoses [7].

(7) Device studies on the pen-injector platform used in the reference injectable semaglutide products have shown accurate dose delivery, low injection force, and favorable usability [21-24].

Cold-chain integrity is equally important:

(1) Injectable semaglutide requires refrigerated storage at 2-8°C before first use.

(2) It must not be frozen.

(3) Inadequate cold-chain maintenance may lead to loss of potency, reduced stability, degradation or aggregation, and altered clinical performance [1,25,26].

Thus, for injectable semaglutide, product quality depends not only on manufacturing but also on device reliability, safe presentation, cold-chain maintenance, and supply-chain traceability.

E: Education/Evidence matters: Similar is not the same

**Figure 7:**
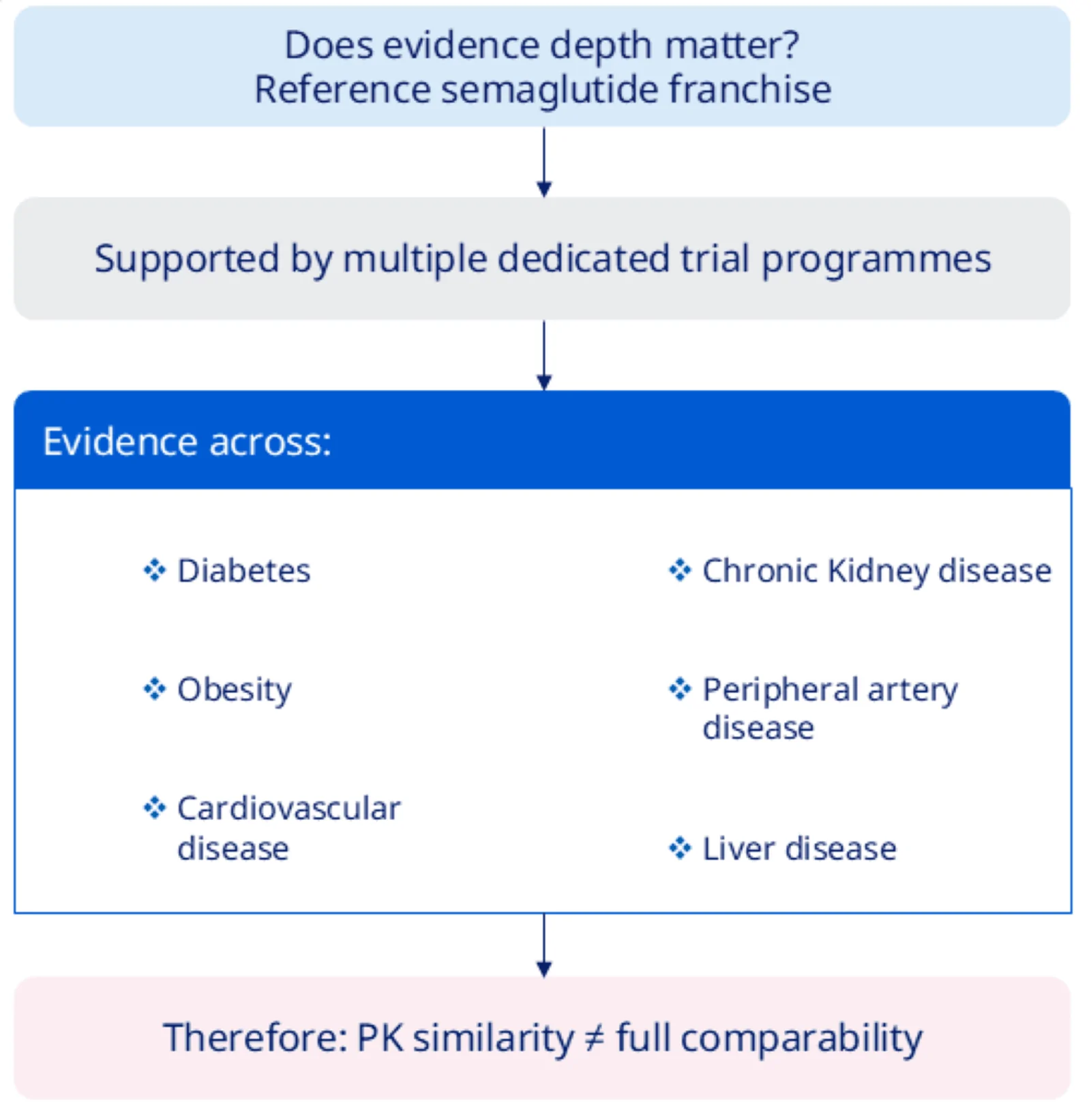
E - Education/Evidence matters: Similar is not the same. Image created by the authors using Microsoft PowerPoint (Microsoft Corporation, Redmond, WA).

Confidence in the original semaglutide franchise derives from the depth, continuity, and breadth of its product-specific evidence base. The reference semaglutide products are distinguished by clinical evidence spanning the cardio-kidney-liver-metabolic (CKLM) spectrum (Figure 7).

Key Evidence

Key evidence includes:

PIONEER and SUSTAIN trials: Established glycemic efficacy and weight reduction across oral and injectable semaglutide in type 2 diabetes [27-29]

STEP trials: Demonstrated substantial weight reduction in obesity [23,30]

SELECT trial: Showed cardiovascular risk reduction in people with overweight or obesity and established cardiovascular disease without diabetes [31]

FLOW trial: Demonstrated kidney benefit in people with type 2 diabetes and chronic kidney disease [32]

SOUL trial: Expanded cardiovascular outcomes evidence for oral semaglutide in people with type 2 diabetes and established atherosclerotic cardiovascular disease and/or chronic kidney disease [33]

STRIDE trial: Extended the vascular evidence by showing improved walking capacity and functional outcomes in people with type 2 diabetes and symptomatic peripheral artery disease [34]

ESSENCE and REMODEL trials: Further broadened the evidence base into metabolic dysfunction-associated steatohepatitis and renal mechanistic pathways, respectively [35-37]

Together, these programs show that confidence in reference semaglutide is grounded in a large, continuous, and product-specific body of evidence spanning glycemic control, weight reduction, cardiovascular protection, kidney outcomes, peripheral vascular disease, and liver-related benefits.

Clinical Implication

Similar naming should not be equated with similar evidence.

A consequence of this evidence pattern is that off-target outcomes, including cardiovascular event reduction (SUSTAIN-6, SELECT, SOUL), kidney outcomes (FLOW), peripheral vascular function (STRIDE), and hepatic outcomes (ESSENCE), have been demonstrated only for the reference product to date. This reflects an absence of dedicated outcome trials for follow-on products [11].

Practical checklist for distinguishing semaglutide products

A semaglutide product should be considered meaningfully comparable to the reference franchise only if the answer is “yes” to five clinically relevant questions captured in the ABCDE checklist (ABCDE is an author-developed conceptual framework): A, is it the same approved active ingredient form; B, has appropriate bioequivalence or clinical bridging been demonstrated; C, is there a regulator-reviewed peptide-specific chemistry, manufacturing, and control strategy; D, is the device and dose-delivery system validated to minimize dosing errors in routine use; and E, is the product sourced through traceable, GDP-aligned distribution? Taken together, these five domains translate the broader scientific principles of identity, evidence, manufacturing consistency, device reliability, and end-to-end product integrity into a practical framework for clinical decision-making. Only when all five criteria are satisfactorily addressed should a semaglutide product be regarded as meaningfully comparable to the reference franchise (Table 2).

**Table 2:** ABCDE* checklist (author-developed conceptual framework) for distinguishing semaglutide products. *ABCDE is an author-developed mnemonic and conceptual framework that has not been endorsed by any regulatory authority. The authors propose that only when these five criteria are satisfactorily addressed should a semaglutide product be considered meaningfully comparable to the reference franchise.

ABCDE checklist		Checklist question	Why it matters
A	Approved active ingredient form	Is it the same active ingredient form as the approved reference product?	Identity should match the approved active moiety, not an alternative or unreviewed salt form.
B	Biopharmaceutical/bridging evidence	Has bioequivalence, or appropriate clinical bridging, been demonstrated?	Clinical trust should come from demonstrated comparability, not presumed similarity.
C	Chemistry, manufacturing, and control strategy	Is there a regulator-reviewed quality dossier with an appropriate peptide-specific manufacturing and control strategy?	Impurity characterization, specifications, comparability, and process control are essential for complex peptide products.
D	Device and dose-delivery reliability	Is the delivery system designed and validated to reduce dosing errors in routine self-administration?	Device presentation affects dose accuracy, usability, and real-world safety.
E	End-to-end traceability (Evidence/Education)	Is the product sourced through traceable, GDP-aligned distribution?	For refrigerated injectable products, storage, transport, and supply-chain integrity are part of product quality.

Although lower-cost synthetic copies of semaglutide products may improve affordability, the originator remains the most extensively characterized option, supported by the broadest clinical evidence base, established manufacturing controls, standardized delivery systems, and mature pharmacovigilance. Accordingly, where access and affordability are not limiting, the originator may reasonably be preferred, while synthetic copies should be considered on the basis of demonstrated quality, regulatory approval, traceability, usability, and continuity of supply rather than price alone, with the support of the author-proposed ABCDE checklist [4,5].

Conclusions

In an increasingly crowded semaglutide landscape, clinical trust should be grounded in the ABCDE framework (author-proposed checklist): approved active ingredient form, biopharmaceutical or bridging evidence, chemistry, manufacturing, and control (CMC) rigor, device reliability, and end-to-end traceability. Using these proposed criteria as a discussion framework, the regulator-approved reference semaglutide products currently have the most complete published body of evidence on identity, quality, clinical outcomes, and device performance among semaglutide products available today. Until alternative products can demonstrate comparable identity, quality, evidence, and usability, many clinicians may reasonably continue to treat the reference products as a useful point of comparison, pending further product-specific evidence on alternatives, rather than assuming equivalence based on sequence or label alone. In routine practice, the choice between the originator and a pharmacosimilar should therefore be approached through informed decision-making and shared decision-making between clinician and patient, with explicit attention to identity, evidence, manufacturing, device performance, traceability, cost, and access rather than name alone.
